# Causal Relationship Between Body Mass Index and Risk of Otitis Media with Effusion in Children: A Mendelian Randomization Study

**DOI:** 10.1007/s12070-023-04161-x

**Published:** 2023-08-31

**Authors:** Jingwen Cao, Wei Liu, Zixuan Yang, Gaoya Qu, Cuiping Zhong

**Affiliations:** 1 Otolaryngology Head and Neck Surgery, The 940th Hospital of the Joint Logistics Support Force of the Chinese People’s Liberation Army, Lanzhou, China; 2 Ningxia Medical University, Yinchuan, China; 3 Key Laboratory of Stem Cells and Gene Province, Lanzhou, China

**Keywords:** Childhood body mass index, Otitis media with effusion, Mendelian randomization, obesity

## Abstract

**Background:**

Body mass index(BMI) in children appears to be associated with Otitis media with effusion(OME) in observational studies, but the causal relationship is not clear.

**Methods:**

A two-sample Mendelian randomization (MR) study was used to explore the causal relationship between childhood BMI and OME in people of European ancestry. Genome-wide association studies (GWAS) of childhood BMI were used as exposures (n = 61,111), while GWAS of OME were used as outcomes (n = 429,290). The weighted inverse variance method (IVW) was used as a baseline method to test for causality. In addition, MR-Egger, simple mode analysis, weighted median, and weighted mode were used as complementary methods.MR-PRESSO analysis, MR-Egger intercept analysis, and Cochran’s Q statistical analysis were also used to detect possible directional heterogeneity and polymorphism. To assess this association, we used ratios (OR) with 95% confidence intervals (ci). All statistical analyses were performed in R.

**Results:**

We selected 22 genome-wide significant single nucleotide polymorphisms (SNPs) from GWAS as instrumental variables (IVW). the IVW approach showed evidence supporting a causal relationship between BMI and OME in children (β = 0.265, SE = 0.113, P = 0.018). MR-Egger regression showed that targeted polymorphisms were unlikely to bias the results bias (intercept=-0.022; P = 0.488), but there was no causal relationship between BMI and OME (β = 0.584, SE = 0.465, P = 0.224). Although the results of the IVW and MR Egger analyses were not consistent, the IVW analysis maintained higher precision, and the Cochran Q test, heterogeneity and polymorphism tests showed no heterogeneity, no directionality and no polymorphism.

**Conclusions:**

MR studies suggest that genetically predicted body mass index in childhood is associated with an increased risk of OME. Notably, given the limitations of this study, the mechanism of association between body mass index and OME in childhood needs further investigation. These results support the importance of effective management of obesity, which may reduce OME occurrence and decrease OME recurrence.

## Introduction

Otitis media(OM) is a common inflammatory disease of the middle ear that presents with pain, fever, anorexia, and irritability, and is one of the leading causes of hearing loss in children [1]. Otitis media with effusion (OME) can occur at any age but is most common between the ages of 6 months and 4 years [2]. Approximately 23% of infants had experienced at least one episode of OM, while 60% of children under three years of age had at least one experience of OM, with 24% experiencing at least three [3]. The most common OM in children is Otitis media with effusion (OME), in which exudate accumulates in the middle ear as fluid and can lead to hearing damage that, if repeated, can affect a child’s hearing and speech development [4]. In addition to known risk factors for otitis media, such as craniofacial anomalies, prematurity, low birth weight, and smoking, there is evidence that obesity may be associated with a high incidence of otitis media [5] .

It is well known that overweight and obese are risk factors for several health problems. Pediatric obesity is a major global problem [6]. According to the World Health Organization (WHO), there are approximately 41 million children under the age of 5 who are obese or overweight worldwide [7]. In the United States, about 32% of children are obese or overweight, and about 17% of these children meet the criteria for obesity [8]. Obesity is a metabolic disease that can lead to low-grade chronic inflammation throughout the body. The International Body Mass Index is known as the Body Mass Index (BMI). Children with an abnormally high BMI are more likely to have complications such as T2D, cardiovascular disease, fatty liver and OSAS [9]. Based on current knowledge, high body fat percentage in children may be a risk factor for developing OME. An analysis of a large number of school-aged children in the United States found that obese children were at significantly higher risk of developing OME than the average child. [10]. Many studies have shown that children with OME have significantly higher average body fat percentages than patients without OME, suggesting that childhood obesity may contribute to the development of OME.

Mendelian randomisation (MR) is a method for assessing causal relationships between risk factors and disease that uses genetic variation as an instrumental variable for risk factors and uses genetic instrumental variables (IVs) to examine potential causal relationships between exposures and outcomes. [11]. As genetic variation occurs randomly by conception and is not influenced by lifestyle or environmental factors, MR analysis minimises potentially unmeasurable confounding factors. In this study, we aimed to use MR analysis to examine the causal relationship between BMI and OME in children.

## Materials and Methods

### MR Design and Data Source

**Fig. 1:**
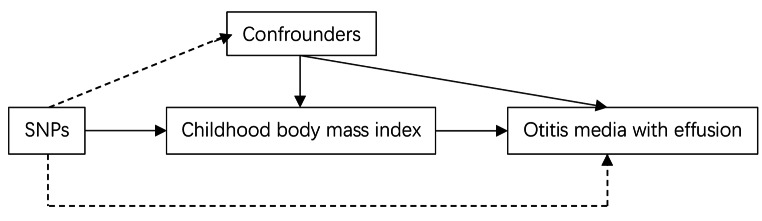
Mendelian randomization model of childhood body mass index and Otitis media with effusion risk in children This design assumes that genetic variants are associated with child body mass index but not with confounding factors and that genetic variants affect Otitis media with effusion only through child body mass index.SNP denotes single nucleotide polymorphism

The authors state that all supporting data are included in this paper and all genomic databases used for the data have been ethically reviewed.The overall structure of this MR study is shown in Fig. 1. We used a two-sample MR model to assess the causal effect of child BMI on OME. MR models are a way to test whether exposure has a causal effect on disease progression, where genetic variation is considered an instrumental variable. The MR approach overcomes undetectable confounders and has a strong ability to make causal inferences [11]. The MR design is based on three assumptions: (1) genetic variation is strongly associated with exposure; and (2) genetic variation is independent of other confounding factors; (3) genetic variation is associated with outcome through exposure only. Eligible exposure and outcome datasets were searched in publicly available databases of genome-wide association studies. Appropriate ethical approval and informed consent were obtained from patients in the initial study, so no further ethical approval was required. We restricted the genetic background of the MR study population to individuals of European descent because population admixture would bias the estimates.

### Body mass Index GWAS Dataset

Genetic instrumental variables for childhood BMI from a publicly available genome-wide association study (GWAS) dataset [12]. The dataset consisted of 26 genome-wide association studies with a meta-analysis of BMI in 61,111 children aged 2–10 years. Finally, we selected 25 SNPs that reached genome-wide significance (P < 5 × 10 − 8) and excluded those with linkage disequilibrium (R2 > 0.1) to screen for 22 independent instrumental variables. The strength of the instrument variables is captured by the F-statistic, which is determined by the proportion of variance explained by the instrument variables (R2). Palindromic SNPs with mean allele fraction response frequency have been excluded from the pool of instrumental SNPs selected (Palindromic SNPs are SNPs with A/T or G/C alleles with a ‘mean allele frequency’ of 0.01–0.30). SNPs with allele frequencies < 0.01 were also removed from the first genomic association study due to their low confidentiality.

### Otitis Media with Effusion GWAS Dataset

Summary data for the association between 22 SNPs associated with childhood BMI and OME were obtained from the recently published genetic variants associated with OME in the GWAS database of the Fingen Institute (https://www.finngen.fi/en). The database contains 429,209 Europeans (9370 cases), identified by the Finnish National Health Registry. The diagnosis of OME is made according to the International Classification of Diseases, 10th revision (ICD-10) and 9th revision (ICD-9) (ICD-10:H65; ICD-9:381). There was no duplication between this sample and the Child BMI study and the majority (98.6%) were of European origin. Each SNP correlation between childhood BMI and OME is shown in Table 1.

**Table 1:**
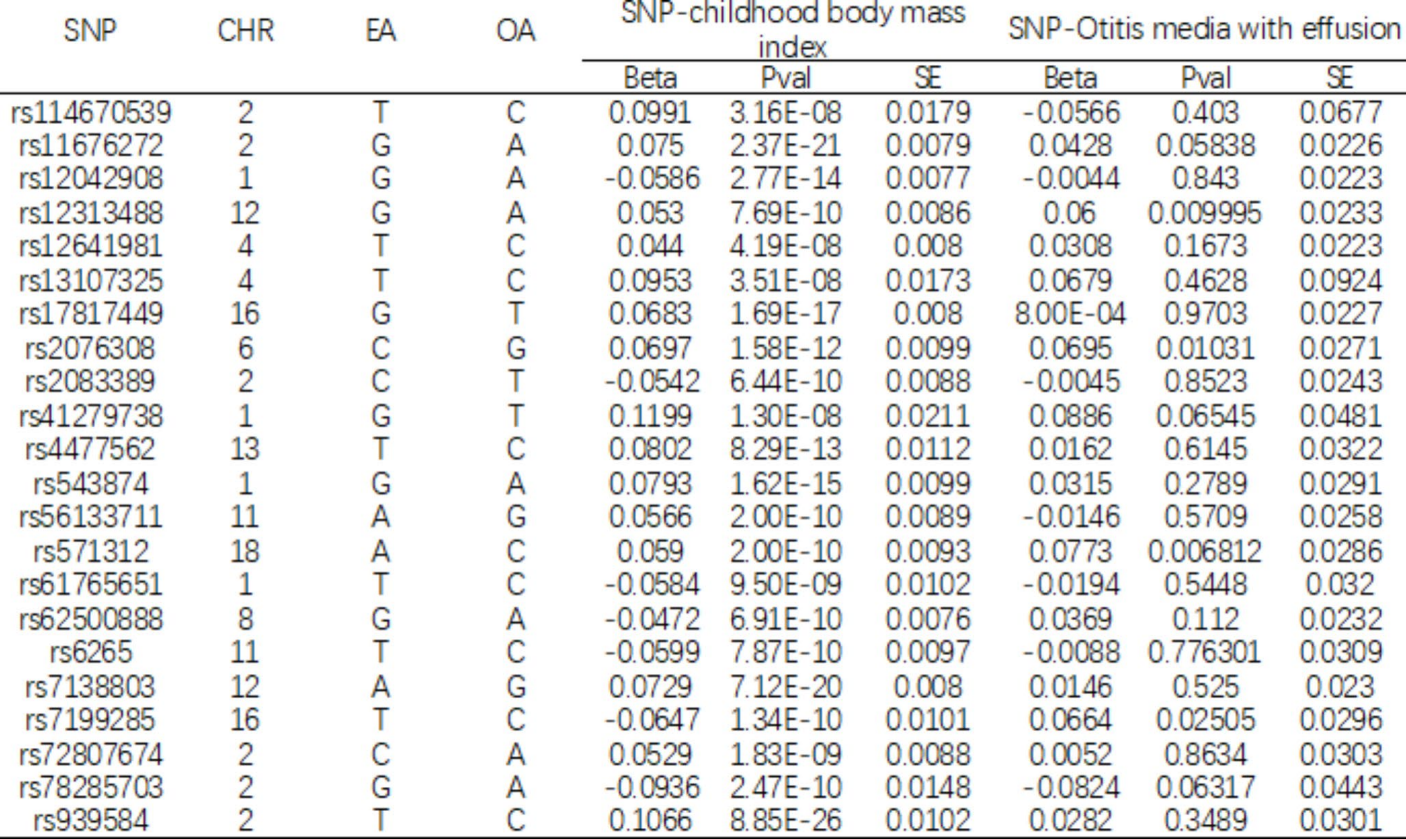
The characteristics of 22 SNPs and their genetic associations with childhood body mass index and Otitis media with effusion SNP, single nucleotide polymorphism; Chr, chromosome; EA, effect allele; OA, other allele; EAF, frequency of effect allele; SE, standard error

To satisfy the first assumption, we selected snp for child BMI at a genome-wide significance level (P < 5 × 10 − 8). snp independency was ensured by linkage disequilibrium (LD) (LD R2 < 0.1, distance LD > 5000 kb). Extract R2 from the original dataset or calculate R2 to indicate the proportion of phenotypic variation explained by IVs. [13]. The SNP statistic F was calculated to determine the strength of the instrument. SNPs with an F-statistic of less than 10 were classified as weakly intense and therefore excluded, ensuring that weak instrumental errors did not affect MR estimates.

## Statistical Analysis

**Fig. 2:**
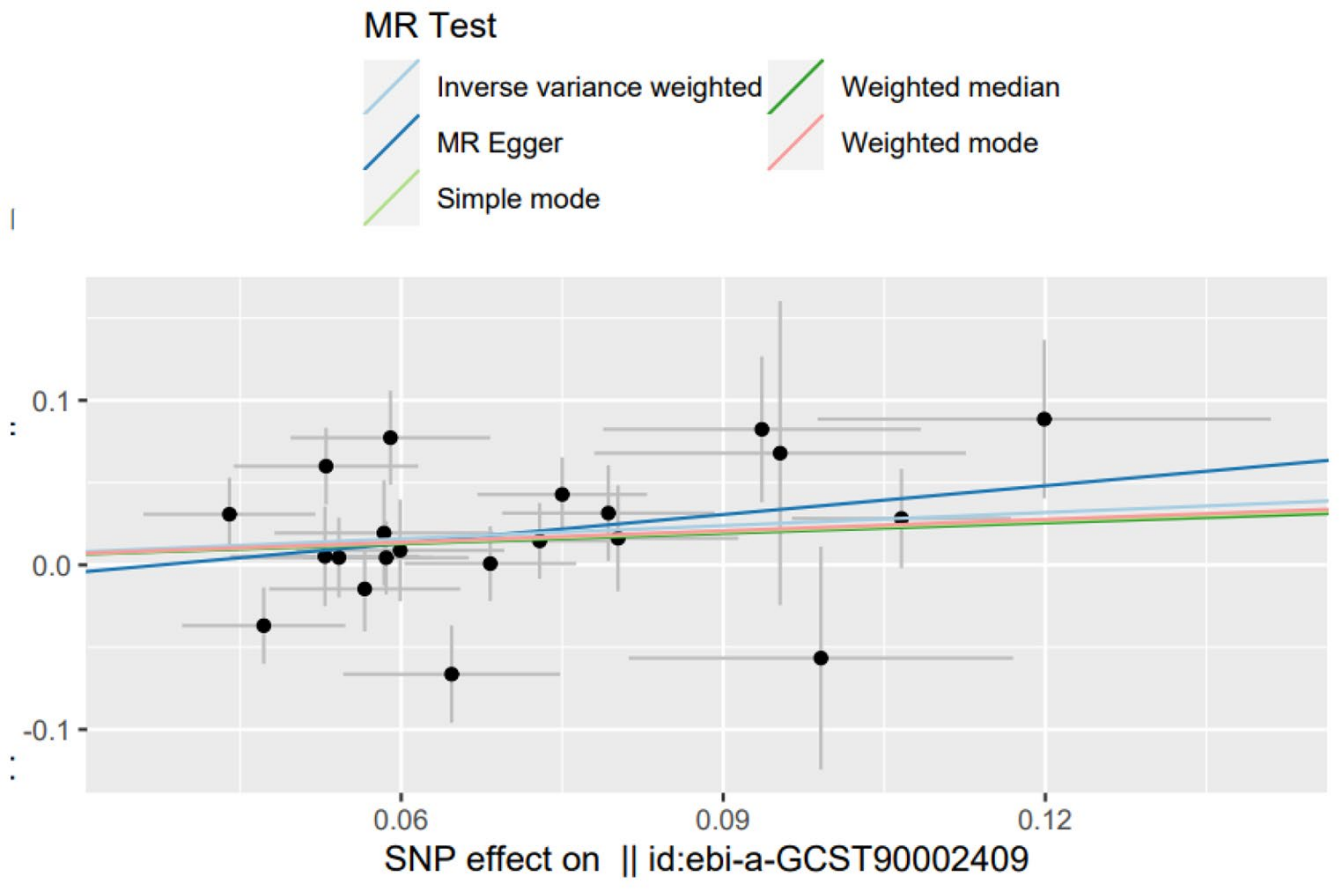
Scatter plot of the effect of childhood body mass index on Otitis media with effusion. The slopes of the solid lines denote the magnitudes of the associations estimated from the MR analyses. MR, Mendelian randomization; SNP, single-nucleotide polymorphism

Two-sample MR analysis was performed using R software (version 4.2.2, R Foundation for Statistical Computing, Vienna, Austria), TwoSampleMR (version 0.5.6), and the MR- presso package (version 1.0.0). In univariate MR, we derived potential causal relationships between the exposure and the outcome using different methods based on different hypotheses: inverse variance weighting (IVW), MR-Egger, weighted median (WME) and Mendelian random multi-effects residuals and outliers (MR-presso). [14]. In the IVW approach, a mate analysis of the Wald association for each of the IVs was conducted to examine causal relationships. [15] The IVW approach assumes that all included genetic variants are effective IVs, while the MR-Egger approach provides a relatively robust estimation that is independent of the validity of the IVs and scales the results according to the available levels of slope and intercept of the multiple regression. [16] WME provided robust results when more than 50% of the weights were from invalid IVs; WME reduced type I errors to assess causality more accurately when cross-sectional polymorphisms were present; and the WME approach provided robust overall causal estimates when the majority of similar individual estimates were from valid IVs. [17] However, the WME, ME and MER approaches are less informative compared to the IVW approach, as shown by the wide confidence intervals (CI), and should only be used in this study as a complementary approach. Heterogeneity was tested with Cochrane Q values. [18]P < 0.05 was considered significant heterogeneity and a random effects model was used for subsequent analyses, otherwise a fixed effects model was used. stability of MR results was determined by excluding IVs one by one and by using omission sensitivity tests. The effect of each of the included IVs on causality was treated by the leave-one-out method [19], and generated scatter plots to visualize the MR analysis results (Fig. 2).

## Results

Two-sample MR analyses were employed to examine the causal relationship between BMI and OME in children. In addition, F > 10 for all these IVs suggests that small instrumental biases do not materially affect causal estimates.

**Fig. 3:**
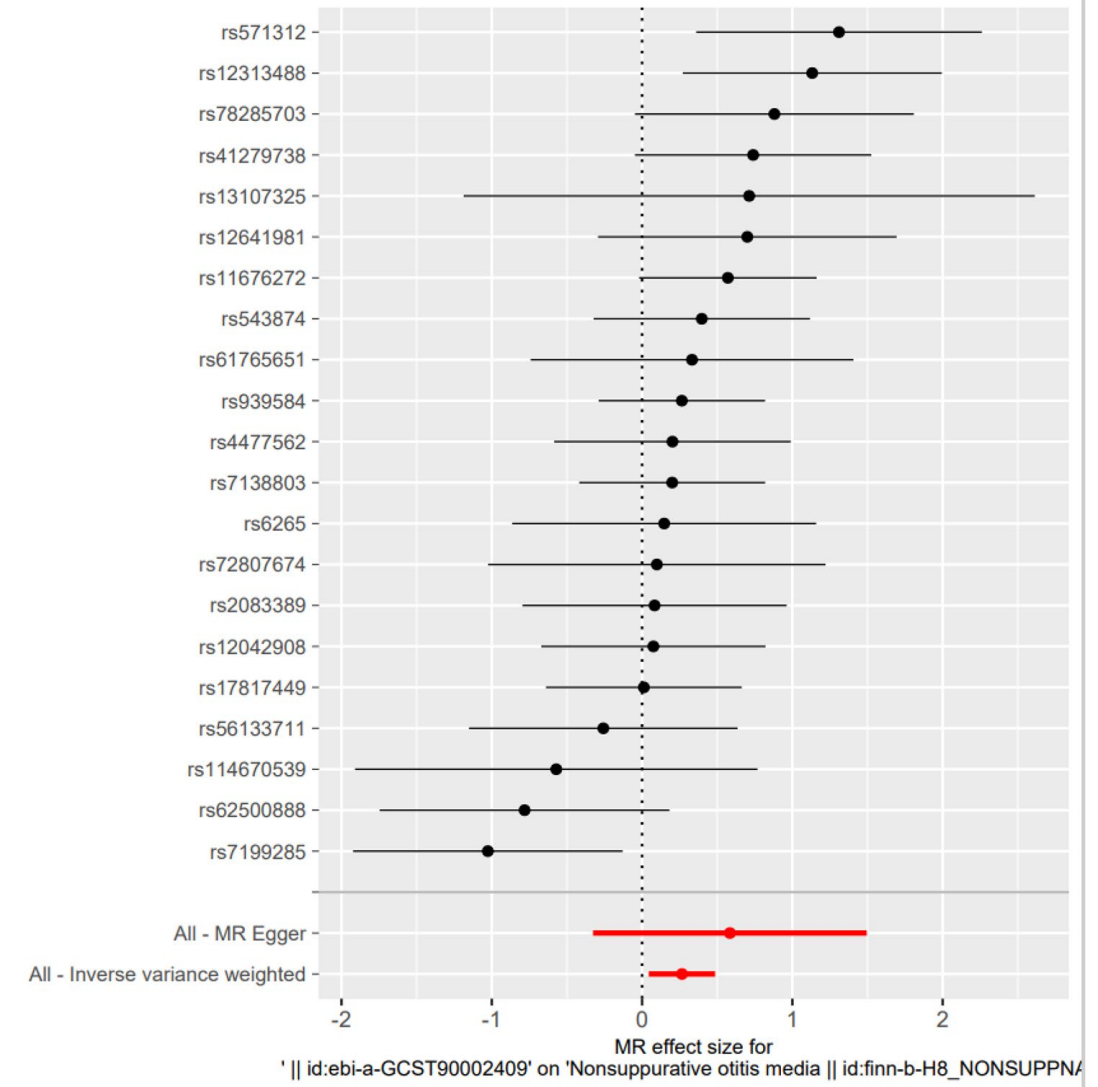
Fixed-effects IVW analysis of the causal relationship between childhood body mass index and Otitis media with effusion in children. The black dots and bars indicate the causal estimate and 95% CI using each SNP. The red dot and bar indicate the overall estimate and 95% CI meta-analyzed by the fixed-effect IVW method IVW, inverse-variance weighted; HI, hearing impairment; CI, confidence interval; SNP, single nucleotide polymorphism

**Fig. 4:**
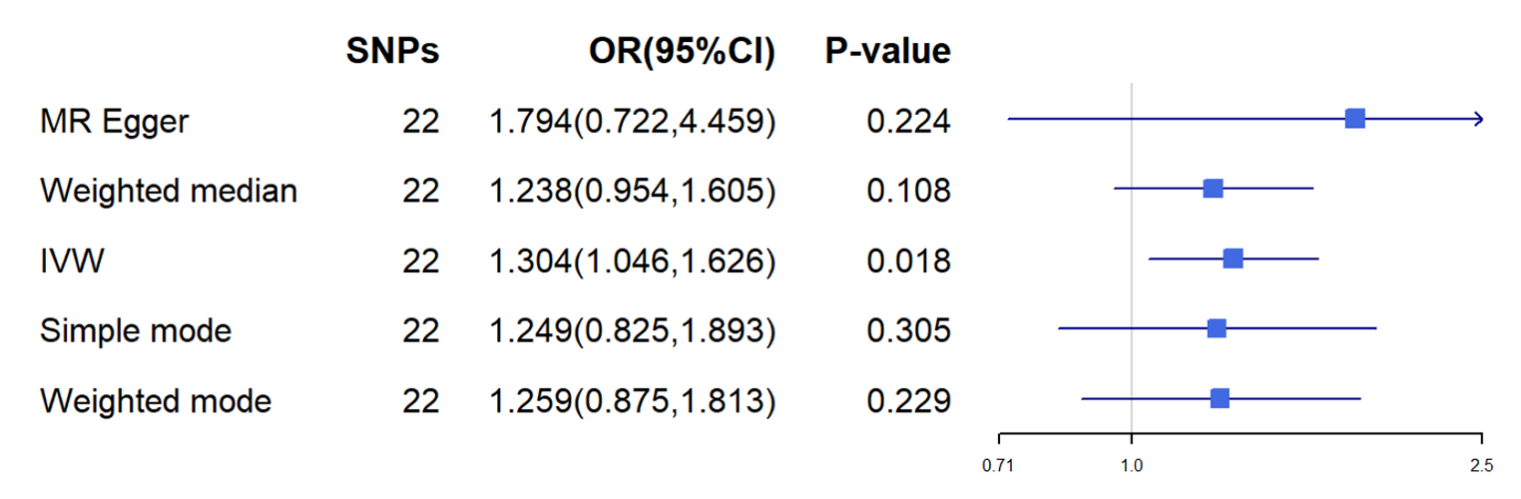
Relationship between BMI and OME in children. The ratio indicates the relationship between the increase in the standard deviation of BMI and OME in children. The IVW random effects method was used as the main analysis method, and other methods were used for sensitivity analysis MR-PRESSO did not find an isolated snp; Abbreviations: IVW = inverse-variance weighted; MR = Mendelian randomization; OR = odds ratio; SNPs = single nucleotide polymorphism

**Fig. 5:**
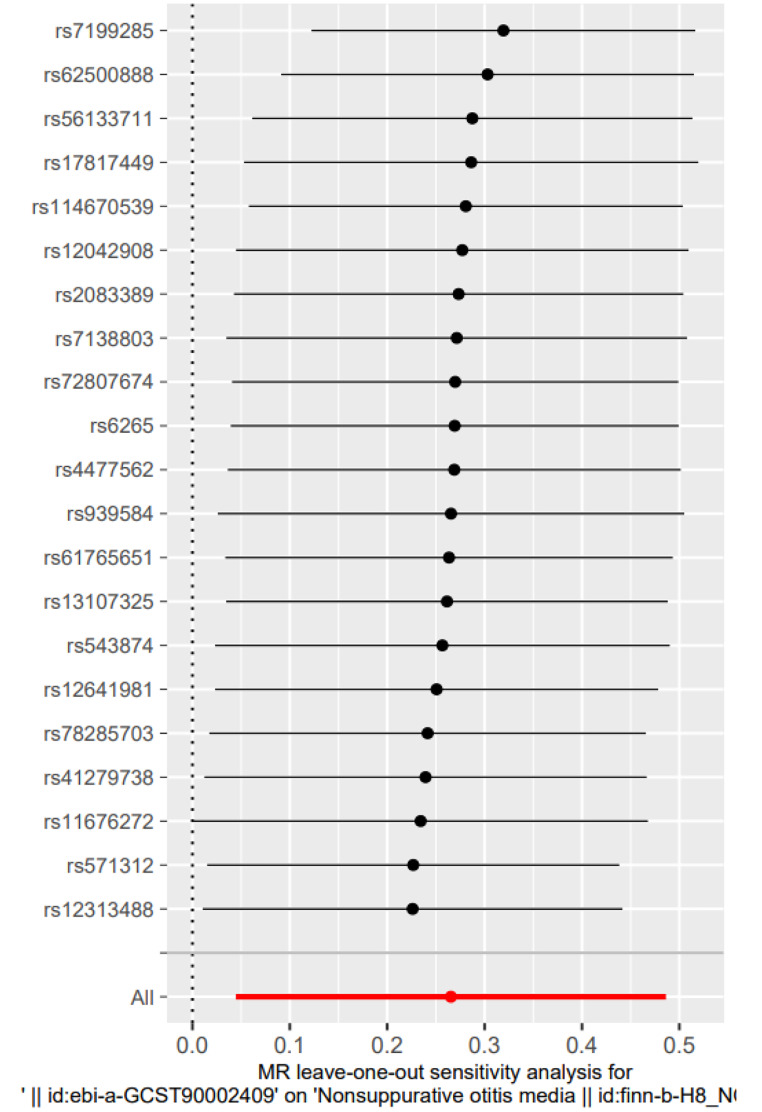
“Leave-one-out” analysis of the causal association of childhood body mass index and Otitis media with effusion.The black dots and bars indicate the causal estimate and 95% CI when an SNP was removed in turn. The red dot and bar indicate the overall estimate and 95% CI using the fixed-effect IVW method; CI, confidence interval; SNP, single nucleotide polymorphism; IVW, inverse-variance weighted

**Fig. 6:**
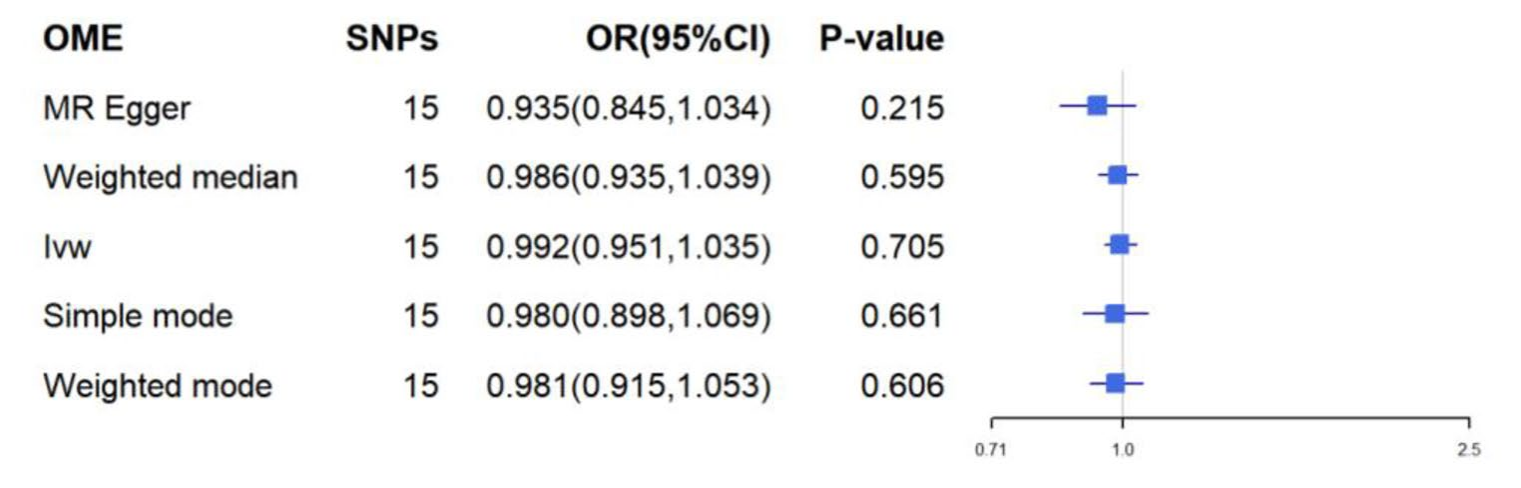
Relationship between OME and BMI in children MR-PRESSO did not find an isolated snp; Abbreviations: IVW = inverse-variance weighted; MR = Mendelian randomization; OR = odds ratio; SNPs = single nucleotide polymorphism

There was no evidence of heterogeneity in Cochran’s Q test (Q = 30.26299, p = 0.06570289), so a fixed effects model was used in the preliminary MR analysis. The IVW analysis revealed a significant causal relationship between child BMI and OME (OR = 1.304, 95% CI [1.089, 1.560], p = 0.004) (Fig. 3), in addition to WME (OR 1, 238, 95% CI [0.954, 1.606], p = 0.108), WM (OR 1.259, 95% CI [0.898, 1.766] p = 0.196) were also comparable (Fig. 4). The MR test was reliable according to the results of the leave-one-out analysis (Fig. 5).No polymorphism (P = 0.488) or heterogeneity (P = 0.066) was found between childhood obesity and OME risk in the MR-PRESSO test and the test of heterogeneity (Table 2). Instead, we reversed the exposure-outcome relationship so that OME was not statistically significantly associated with child BMI when used as an exposure measure (Fig. 6).

**Table 2:**
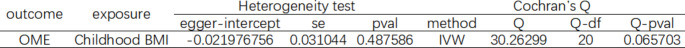
Results of polymorphism and heterogeneity tests for the association between BMI and OME in children

## Discussion

This study extends the epidemiological evidence by demonstrating a potential causal relationship between genetically predicted high BMI and OME using large-scale genetic data within an MR framework. We used three different estimation methods in our MR analysis (inverse variance weighting, weighted median method and MR-egger regression). Our study showed that to eliminate polymorphism, we used weighted median estimation, which provides valid estimates even if 50% SNP is not a valid instrument, and we used MR-EGGER regression to provide a test for unbalanced polymorphism, as well as to expose causal estimates of the outcome in the presence of polymorphism [17]. There may be a causal relationship between BMI and OME in children. Although MR estimates using IVW, MR-Egger, and weighted median analyses were inconsistent, IVW analyses supported a causal relationship between BMI and OME in children. Considering that IVW has the advantage of maintaining a higher estimation precision compared to MR-Egger analysis [20], This MR analysis suggests a potential causal role of childhood BMI in the risk of OME prevalence. Thus, our study confirms the correlation found in previous observational studies.

According to current knowledge, there is an association between obesity and both endocrinology and immunology [21]. However, the biological mechanisms by which obesity is associated with OME are unclear. Obesity may contribute to OME through the following mechanisms: alterations in cytokine levels, such as IL-6, TNF-α, and fibrinogen activator inhibitor-1 (FAI-1); alterations in host immunity; induction of gastroesophageal reflux; and alterations in pharyngeal tube structure [22–24]. In short, adipose tissue interferes with the endocrine and immune systems. A meta-analysis by Cottam et al. found that serum levels of IL-6 and TNF-α were higher in obese patients than in lean patients and that IL-6 was present in the middle ear (ME) fluid of most patients with chronic otitis media [25]. Interestingly, there was no significant difference in total intake between OME and healthy patients, while the difference in fat intake was significant. This suggests that OME is associated with a high-fat diet rather than with a large amount of food [26]. A high-fat diet that leads to impaired immune regulation may increase the incidence of respiratory infections and pharyngeal dysfunction, thereby increasing susceptibility to OME [27]. Also, a high-fat diet that aggravates GERD can lead to OME [28].

In comparison, a different argument has been put forward by some researchers that frequent OME may alter taste perception at the front of the tongue by affecting the function of the bulbar nerve [29]. As a result, children with recurrent OME have a low preference for vegetables and fruits, preferring foods high in oil and sugar [30].

Although observational studies have reported an association between obesity and OME, this association may be influenced by environmental confounders. The present study is the first report to extend the epidemiological evidence by using pooled data from a large genome-wide association study to confirm a potential causal relationship between BMI and OME in children. We performed several sensitivity analyses to test this hypothesis. Furthermore, when we adjusted for the relationship between OME and exposure to childhood BMI and outcome, this relationship became statistically insignificant, suggesting that the direction of causality between childhood BMI and OME is not bidirectional.

Our results suggest that the occurrence of OME may be independently influenced by hereditary BMI. Notably, due to potential confounding factors, further studies are needed to replicate our results on BMI and OME. However, our MR analysis has several limitations. First, due to the classification of the raw data, we were unable to further subdivide the types of non-suppurative otitis media, so we could only analyze non-suppurative otitis media as a whole. Second, although Mendelian randomization has been shown to be a robust method for assessing the causal relationship between childhood BMI and OME, two-sample MR analysis provides only an estimate of the hypothesized causal relationship, and further studies are needed to estimate the direct causal effect of childhood obesity on OME. Third, the GWAS 1 data were compiled for individuals of European ancestry, which means that our results may not be fully representative of the entire population. Overfitting and instrumental bias became more pronounced as the overlap between samples increased, similar to what was observed in the single-sample MR study.

## Conclusion

Using a two-sample MR framework, this study provides strong evidence for a potential causal relationship between BMI and genetic susceptibility to OME in children. These results support the importance of effective obesity management, which may reduce the incidence of childhood OME and improve the prognosis of patients with OME. Notably, given the limitations of this study, the mechanism of interaction between childhood BMI and OME requires further investigation.
